# Polymorphism on human aromatase affects protein dynamics and substrate binding: spectroscopic evidence

**DOI:** 10.1186/s13062-021-00292-9

**Published:** 2021-04-26

**Authors:** Giovanna Di Nardo, Almerinda Di Venere, Chao Zhang, Eleonora Nicolai, Silvia Castrignanò, Luisa Di Paola, Gianfranco Gilardi, Giampiero Mei

**Affiliations:** 1 Dipartimento di Scienze della Vita e Biologia dei Sistemi, Università di Torino, Via Accademia Albertina 13, 10123 Turin, Italy; 2 Dipartimento di Medicina Sperimentale, Università di Roma Tor Vergata, Via Montpellier 1, 00133 Rome, Italy; 3 Dipartimento di Ingegneria, Unità di Fondamenti Chimico-Fisici dell’Ingegneria Chimica, Università Campus Bio-Medico di Roma, via Álvaro del Portillo 21, 00128 Rome, Italy

**Keywords:** Aromatase polymorphism, Ligand binding, Fluorescence, Molecular modeling

## Abstract

Human aromatase is a member of the cytochrome P450 superfamily, involved in steroid hormones biosynthesis. In particular, it converts androgen into estrogens being therefore responsible for the correct sex steroids balance. Due to its capacity in producing estrogens it has also been considered as a promising target for breast cancer therapy. Two single-nucleotide polymorphisms (R264C and R264H) have been shown to alter aromatase activity and they have been associated to an increased or decreased risk for estrogen-dependent pathologies. Here, the effect of these mutations on the protein dynamics is investigated by UV/FTIR and time resolved fluorescence spectroscopy. H/D exchange rates were measured by FTIR for the three proteins in the ligand-free, substrate- and inhibitor-bound forms and the data indicate that the wild-type enzyme undergoes a conformational change leading to a more compact tertiary structure upon substrate or inhibitor binding. Indeed, the H/D exchange rates are decreased when a ligand is present. In the variants, the exchange rates in the ligand-free and –bound forms are similar, indicating that a structural change is lacking, despite the single amino acid substitution is located in the peripheral shell of the protein molecule. Moreover, the fluorescence lifetimes data show that the quenching effect on tryptophan-224 observed upon ligand binding in the wild-type, is absent in both variants. Since this residue is located in the catalytic pocket, these findings suggest that substrate entrance and/or retention in the active site is partially compromised in both mutants. A contact network analysis demonstrates that the protein structure is organized in two main clusters, whose connectivity is altered by ligand binding, especially in correspondence of helix-G, where the amino acid substitutions occur. Our findings demonstrate that SNPs resulting in mutations on aromatase surface modify the protein flexibility that is required for substrate binding and catalysis. The cluster analysis provides a rationale for such effect, suggesting helix G as a possible target for aromatase inhibition.

## Background

Structural flexibility is a crucial property of proteins, since it allows the molecular rearrangements required for most of their biological functions. Such rearrangements include large conformational changes (i.e. those characterizing cargo and contractile proteins), long distance displacements (for instance, those occurring in some integral membrane receptors), or even smaller, local conformational changes (as those required for the action of most enzymes). Flexibility allows substrates binding, products release and it is needed to confer allosteric properties to enzymes that control and regulate metabolic pathways and cells growth. Cytochromes P450 are a very good example of such enzymes as they are heme-containing monooxygenases involved in key metabolic pathways and xenobiotic detoxification. Moreover, they are known to undergo conformational changes to allow substrate access, catalysis and product release [1]. In particular, structural data demonstrate that some elements such as the F- and G- helices and the loop connecting them (F-G loop) are highly flexible and can open and close channels connecting the surface of the protein to the active site [2, 3].

Human cytochromes P450 are highly polymorphic and some single nucleotide polymorphisms (SNPs) are known to affect the enzyme activity by decreasing the catalytic efficiency. Although the effect of many SNPs can be rationalized by the location of the mutations in the protein structure, for others it is very difficult to predict. This is the case of two SNPs present in human aromatase, a cytochrome P450 with a key role in steroidogenesis as it catalyzes the conversion of androgens into estrogens [4]. The correct activity of this protein is crucial, because alterations in the equilibrium between androgens and estrogens, hypo- or hyper-production of estrogens are associated to a series of disorders that range from neurological diseases to breast cancer [5]. We have previously characterized the common R264C (rs700519) and the rarer R264H (rs2304462) polymorphic variants showing that the activity of these variants is modified by an alteration of the kinetic parameters [6, 7] and by a different phosphorylation propensity, as R264 is part of a consensus sequence for phosphorylation [6]. However, this residue lies on the protein surface, being part of the G-helix and it is therefore difficult to understand how its mutation can impact the catalytic parameters only based on the location.

We previously demonstrated that aromatase requires dynamic flexibility to bind and process its substrates [8]. In this paper we have used a combination of experimental techniques (namely infrared and fluorescence spectroscopy) to evaluate the impact that a single point mutation (R264C or R264H) on the external surface of human aromatase has on its tridimensional structure and on its biological function. In parallel to such experimental approach, a contact network analysis of the protein structure has been also performed. In the last decade the application of this computational methodology to proteins has allowed to look at these complex molecules from a new perspective, since such kind of algorithms use a few descriptors to characterize the spatial inter-connections among amino acids. In this way, clusters of residues are localized within the protein tridimensional structure and long-range interactions, cooperative effects and segmental mobility can be predicted. The applications of this methodology span from protein-protein interaction [9, 10] to allosteric regulation of enzymes [11, 12], allowing, in very recent researches, to speculate on possible binding sites for drugs against degenerative diseases [13] or viruses [14]. We demonstrate that the combination of this approach with experimental data might suggest a molecular explanation to the loss of biological activity observed in R264C and R264H variants.

## Results

### H/D exchange followed by FTIR

**Fig. 1:**
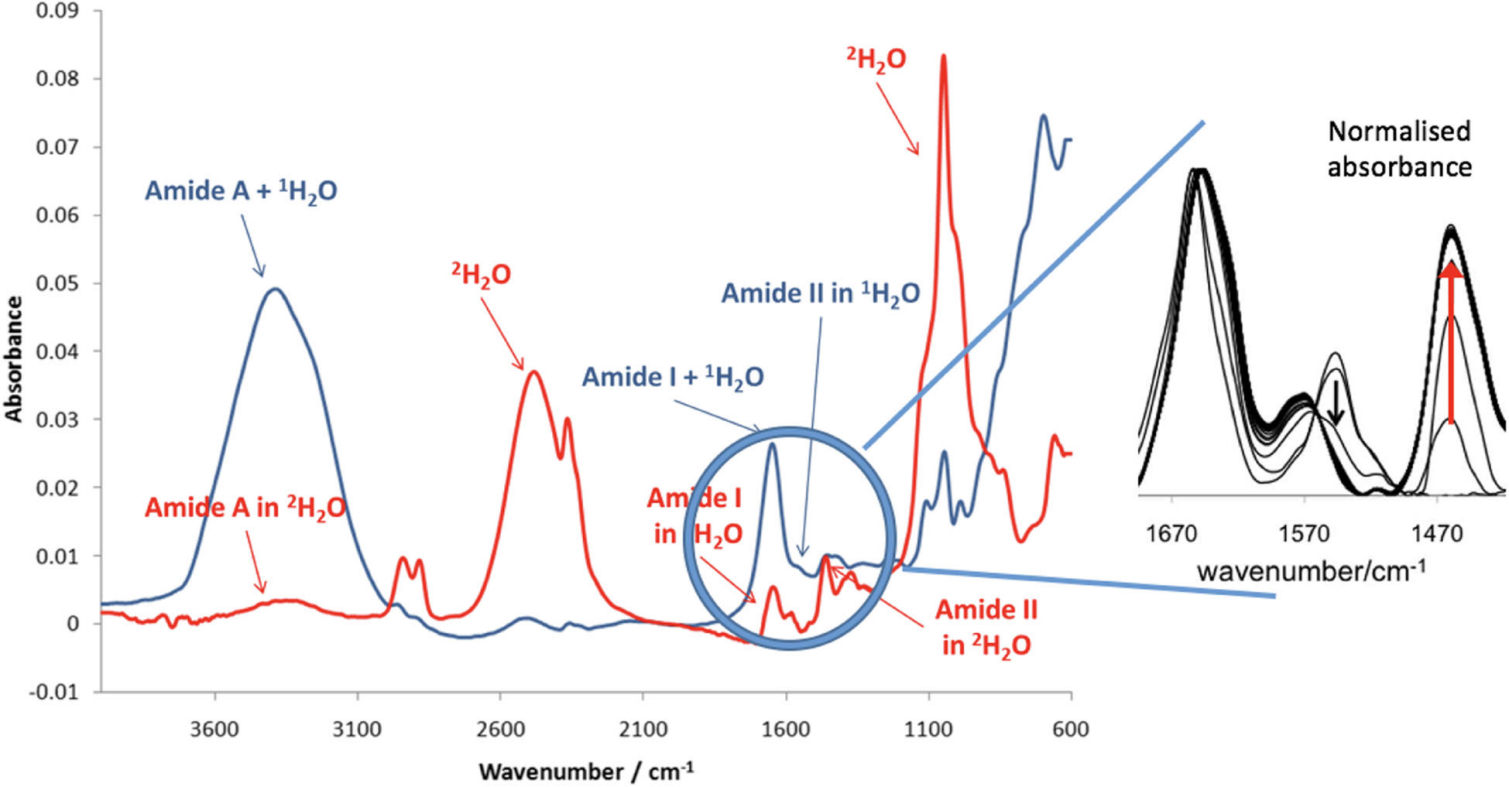
FTIR spectra of apo-Aro at the beginning (blue) and at the end (red) of a deuteration kinetics. In the inset (right side) the increase of the absorption intensity upon deuteration, at λ = 1460 cm^− 1^ is shown (red arrow) after normalization of the spectra at 1650 cm^− 1^

Fourier transform infrared spectroscopy (FTIR) was applied to determine the kinetics of H/D exchange in Recombinant human aromatase (Aro) and its polymorphic variants, to detect possible differences in the kinetics of deuteration arising from protein conformational changes induced by ligand binding. Figure 1 shows the FTIR spectra recorded at the beginning and at the end of a typical H/D exchange kinetic experiment, in the case of substrate-free Aro.

The band between 1670 cm^− 1^ and 1620 cm^− 1^ has been assigned to the amide I, whereas the band between 1470 cm^− 1^ and 1420 cm^− 1^ has been attributed to the amide II. The H/D exchange was thus followed as the increase of the absorbance of the amide II band at 1460 cm^− 1^ (Fig. 1, inset), in a time range of 160 min.

**Fig. 2:**
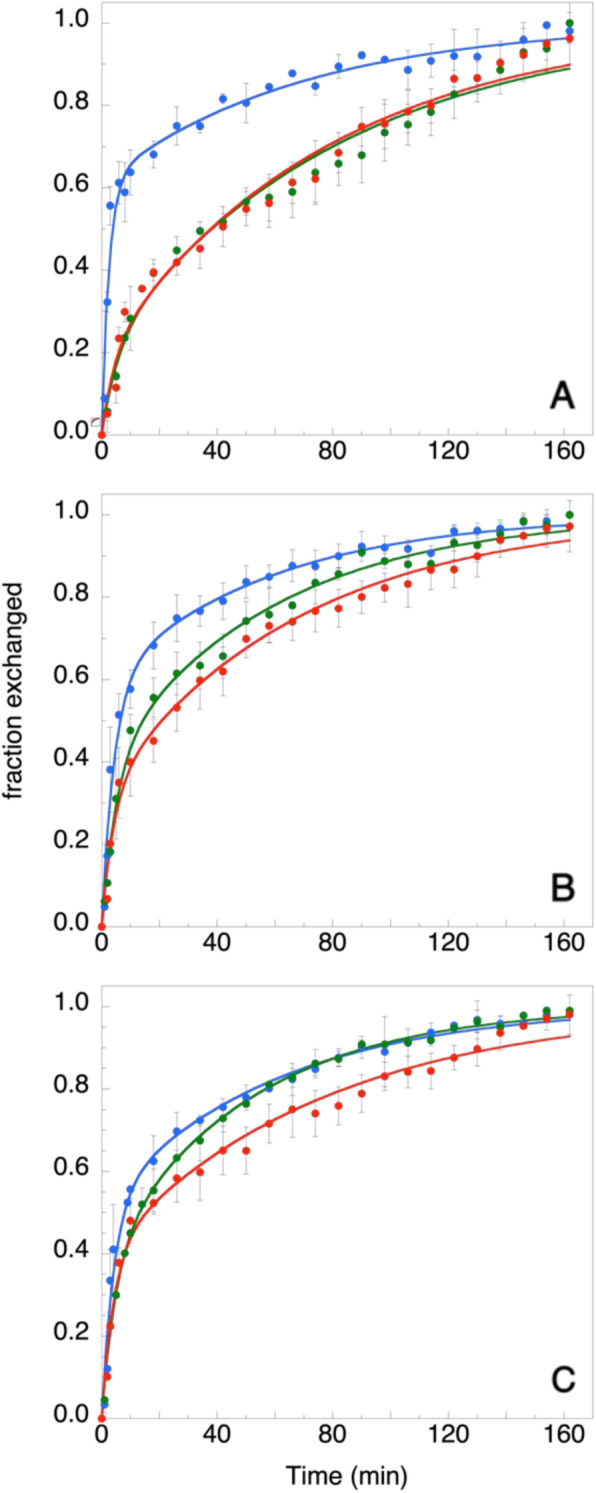
Kinetics of H/D exchange followed by FTIR for wt-Aro (panel **a**, blue symbols) and the two polymorphic variants (namely R264H, panel **b**, and R264C, panel **c**, blue symbols). The kinetics in the presence of the substrate androstenedione (green symbols) and the inhibitor anastrozole (red symbols) are also reported. The solid lines correspond to the best double exponential fitting curves, whose parameters are reported in Table 1

The time dependence of the fraction of protons exchanged shows a dramatic increase in the first ten minutes, while a much slower kinetics takes place in the next 2–3 h (Fig. 2). The data are, in fact, best fitted to a double exponential curve (cfr. Methods), which yields two distinct rate values (Table 1). The Aro wt sample is characterized by the faster initial transition (k_1_ ≈ 0.4 m^− 1^), a process that is considerably slowed down by ligand binding (Fig. 1, panel A), the k_1_ value being reduced to one half both in the presence of the substrate and the inhibitor molecules (Table 1). No significant differences are instead observed in the long-term component (k_2_), indicating that the slower part of the kinetic is independent of the binding process. In the H/D exchange kinetics, unlike Aro wt, the presence of the substrate androstenedione and the inhibitor anastrozole on polymorphic variants is less relevant (Fig. 2b, c). In particular, the raw data are characterized by a slower increase in the initial part of the process (t ≈ 0–20 min, Fig. 2), whose fit yields a lower rate constant (k_1_ ≈ 0.2 min^− 1^, Table 1), with respect to that of the wt protein. Again, the introduction of androstenedione or anastrozole in both mutants does not affect the second step of the kinetics (Fig. 2b, c) and, in fact, no significant changes are observed in the second rate constant value (k_2_ ≈ 0.016 min^− 1^, Table 1).

**Table 1:** Results of the double exponential fits performed on the data reported in Fig. 2

sample	m_1_^(*)^ (min)	k_1_(m^− 1^) (min^− 1^)	k_2_(m^− 1^) (min^− 1^)
Aro wt	0.61 ± 0.03	0.40 ± 0.04	0.014 ± 0.002
Aro wt + sub	0.20 ± 0.05	0.18 ± 0.05	0.012 ± 0.001
Aro wt + inib	0.19 ± 0.03	0.23 ± 0.06	0.012 ± 0.001
R264H	0.59 ± 0.03	0.22 ± 0.05	0.017 ± 0.001
R264H + sub	0.39 ± 0.03	0.19 ± 0.03	0.017 ± 0.002
R264H + inib	0.32 ± 0.02	0.23 ± 0.04	0.015 ± 0.001
R264C	0.52 ± 0.03	0.23 ± 0.05	0.017 ± 0.002
R264C + sub	0.38 ± 0.03	0.20 ± 0.02	0.020 ± 0.001
R264C + inib	0.40 ± 0.03	0.25 ± 0.04	0.013 ± 0.002

^(*)^m_2_ = 1- m_1_

The presence of a complex kinetics (Table 1) indicates a heterogeneity in the protons populations. Indeed, a bi-phasic behaviour of the H/D exchange process in proteins is generally explained in terms of two groups of peptide hydrogens, one more accessible to the solvent molecules (and thus subject to a higher exchanging rate) and a second one located in more buried protein segments, less accessible to water [15–18]. Since the H/D exchange process at the protein surface would be too fast to be detected, the two rate constants must be assigned to partially exposed (k_1_) or buried (k_2_) residues protons. In such a context, a relevant meaning may be attributed to the pre-exponential factors m_i_ of the respective fits, as they represent the initial fractions (i.e. at time = 0) of each protons population. As shown in Table 1, the m_1_ value of the wt protein is the highest (≈ 0.60) in the absence of any ligand, and the lowest (≈ 0.20) when one of the ligands is present, thus indicating that a large change, Δm_1_ ≈ − 65%, occurs in the population of the partially exposed protons upon binding.

On the contrary, the decrease observed in the case of the two mutants results to be smaller (Δm_1_ ≈ − 40% for R264H and Δm_1_ ≈ − 23% in the case of R264C), suggesting less pronounced structural effects induced by ligand binding.

### Intrinsic fluorescence dynamics

**Fig. 3:**
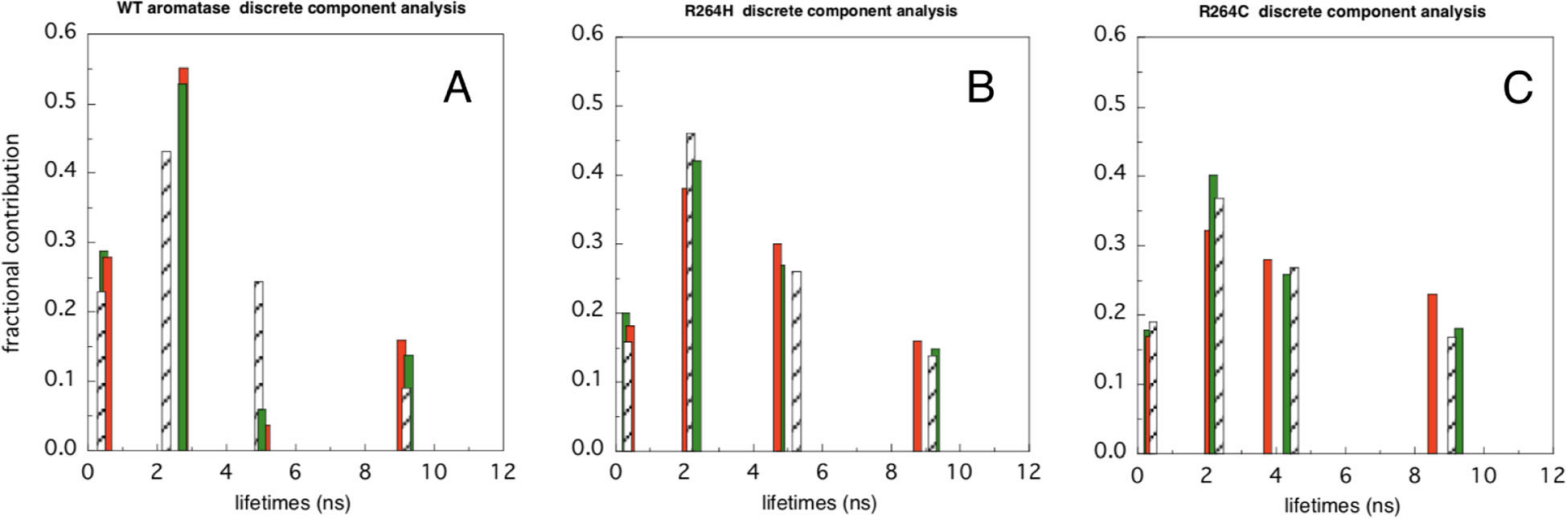
Lifetime components of wt-Aro fluorescence decay (panel **a**), R264H (panel **b**) and R264C (panel **c**) in the absence (dashed bars) and in the presence of substrate (green) or inhibitor (red)

Ligand-induced changes in the intrinsic fluorescence of aromatase have been characterized through the phase-shift and demodulation technique and the results of data analysis are shown in Fig. 3. As previously observed [8], the protein emission decay is quite heterogeneous, being characterized by four distinct lifetime components, ranging from tenths of nanoseconds to 6–9 ns. Such a complexity is due to the presence of five tryptophan residues, located in different environments of the protein scaffolding. The main feature of the wt sample in the presence of either substrate or inhibitor is a large decrease of the fractional contribution at ≈ 5 ns (Fig. 3a). Such a considerable quenching effect has been attributed by previous fluorescence measurements to the interaction between the substrate and a specific tryptophan residue, namely W224, which is buried in the core of the protein matrix [8].

As shown in Fig. 3b and c, no changes were instead detected in the fractional contribution at 5 ns of both R264H and R264C upon ligands addition, indicating that in the mutants the binding of either substrate or inhibitor molecules does not produce significant effect in the environment of W224, suggesting a different local conformation with respect to that of the wt cavity.

**Fig. 4:**
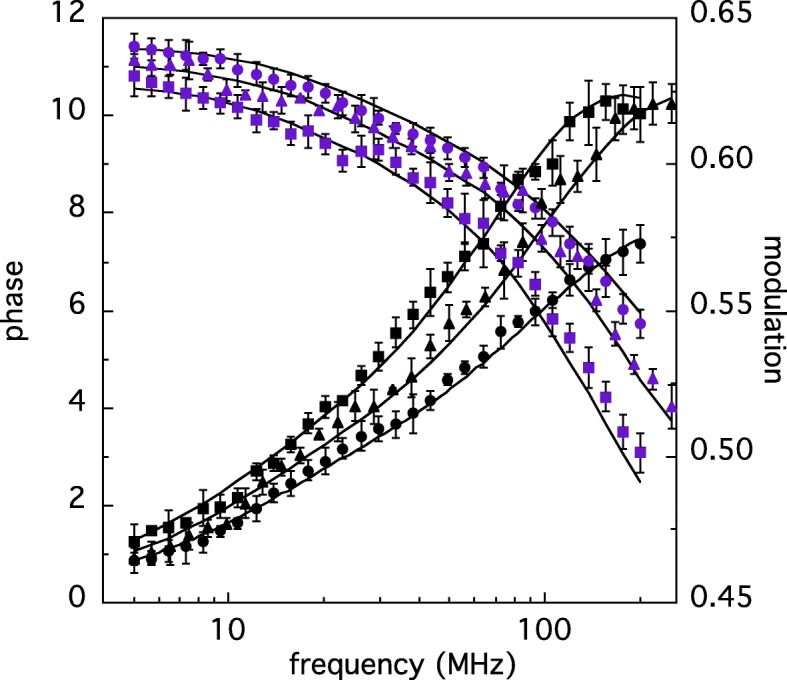
Phase shift (black symbols) and demodulation data (purple symbols) of wt-Aro (circles), R264H (squares) and R264C (triangles). The solid lines correspond to the best fit obtained using two rotational correlation times

This hypothesis has been further tested by studying the protein rotational dynamics. The presence of the long lifetime component, in fact, allows to perform anisotropy measurements using the aromatase intrinsic fluorescence. The corresponding phase shift and demodulation data are reported in Fig. 4, for Aro wt and R264C or R264H mutants. The results demonstrate that both mutated forms display slower rotational dynamics, with respect to the wt protein, the data being shifted toward a lower frequency range.

**Fig. 5:**
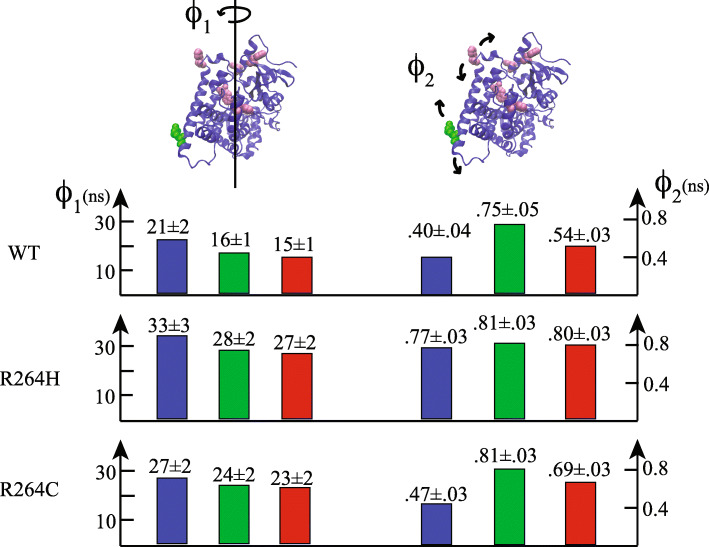
Long (ϕ_1_) and short (ϕ_2_) fluorescence correlation lifetimes of apo-wt and apo-mutants (blue) aromatase. The values of ϕ_1_ and ϕ_2_ upon the addition of substrate or inhibitor are reported in green and red, respectively. In the cartoons, a ribbon model of aromatase is reported in which the positions of R264 and of the five tryptophans are highlighted in green and pink VDW spheres

In fact, the phase and demodulation curves of each aromatase type crosses each other at ≈ 150, 63, and 93 MHz, respectively in the case wt, R264H and R264C, indicating that R264H is the form characterized by the slowest dynamics. Non-linear fitting of the phase shift and demodulation points reported in Fig. 4 yielded the results illustrated in Fig. 5. Each data set required two rotational correlation times, ϕ_1_, and ϕ_2_. The first one, ϕ_1_, ranges from 21 ns (wt) to 33 ns (R264H) and it is compatible with the rotational motion of whole protein, as shown in the cartoon sketched in the left side of Fig. 5. Indeed, the predicted turning on a principal axis of a spherical hydrated molecule with the same size of aromatase (≈ 58,000) would be ϕ_sph_ ≈ 24 ns [19]. Longer ϕ_1_values, such those characterizing R264C and R264H, suggest a more swelled tridimensional form, since an expanded tertiary structure would produce a slower motion.

In the case of the wt sample, the entrance of the substrate (or inhibitor) in the protein binding cavity produced a considerable decrease in the ϕ_1_value (≈ − 23%, Fig. 5), thus implying a faster rotational dynamics. This trend was also observed in the mutated forms but with two important differences: i) the extent of the relative change in ϕ_1_ is less pronounced (≈ − 11% and − 15%, respectively); ii) in both samples, the ϕ_1_ value remains above 23 ns, independently of the ligand (substrate or inhibitor) used (Fig. 5).

The second rotational lifetime,ϕ_2_,is much shorter being on the order of ≈ 400–500 ps, in the case of wt and R264C aromatase and ≈ 800 ps, as regards R264H (Fig. 5). Such values reflect a faster dynamics (with respect to ϕ_1_), that can be ascribed to the average local motions of tryptophan(s) environment. The presence of the ligands in the binding pocket slows down such movements in the case of the wt and the R264C samples, the substrate molecule being the most efficient, since ϕ_2_isalmost doubled in the holo-form (Fig. 5). On the contrary, the addition of the substrate or the inhibitor does not produce significant changes in the local dynamics of R264H, as shown by the similarity of ϕ_2_ the values.

### ANS binding

**Fig. 6:**
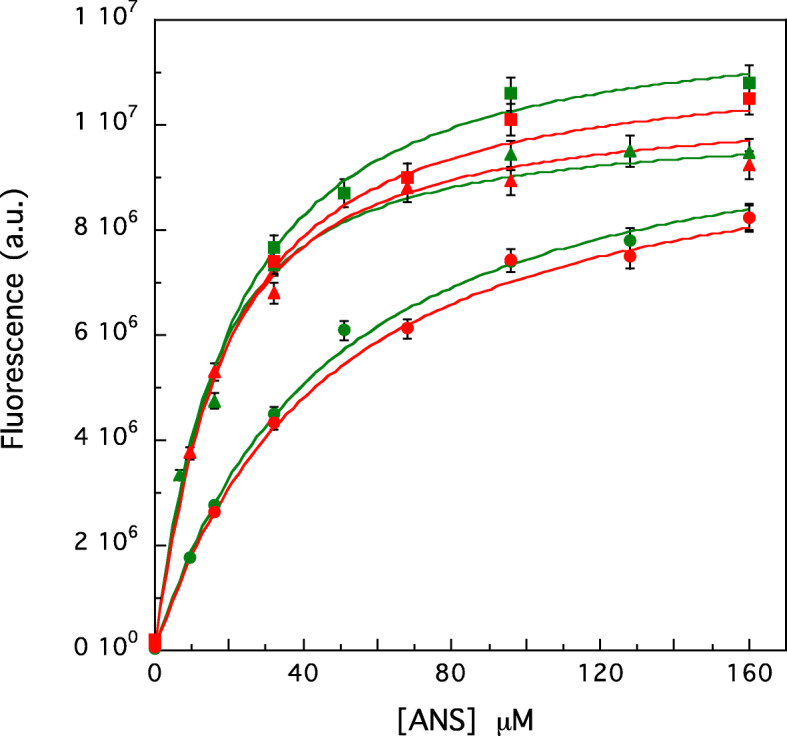
ANS-binding curves of wt-Aro (circles) and mutants R264H (squares) or R264C (triangles) in the presence of saturating concentration of substrate (green) or inhibitor (red). The concentration of all the proteins is 6.4 μM. The solid lines correspond to the best fit obtained for each sample by a chi-squared minimization procedure (see materials and methods)

8-Anilino-1-naphthalenesulfonic acid (ANS) is a hydrophobic probe, which increases its fluorescence intensity upon binding to surface cavities and pockets of proteins [20]. For such reason, it is particularly suitable to study the conformational changes that modify the compactness of a protein tridimensional structure, such as those produced by temperature [21], pH [22], pressure [23] and substrate binding [24]. In order to characterize to what extent the presence of substrate (or inhibitor) in the active site of aromatase alter the roughness and accessibility of the protein surface, we added increasing amounts of ANS to the ligand-containing samples and compared the respective titration curves and fit in Fig. 6. As shown also by the respective dissociation constants (reported in Table 2), the wt protein displays a lower affinity for ANS, its K_d_ value being 3 times larger than that obtained in the case of R264H and 2 times with respect to R264C (Table 2). Interestingly, in all samples the ANS binding process does not depend on the ligand used, giving the same results for the substrate and the inhibitor (Fig. 6 and Table 2).

**Table 2:** ANS-aromatase dissociation constants yielded by the fits reported in Fig. 6

Sample	K_d_ (mM)
Aro wt + sub.	40 ± 2
Aro wt + inh.	41 ± 3
R264H + sub.	11 ± 1
R264H + inh.	13 ± 1
R264C + sub	17 ± 2
R264C + inh.	16 ± 2

### Contact network analysis

**Fig. 7:**
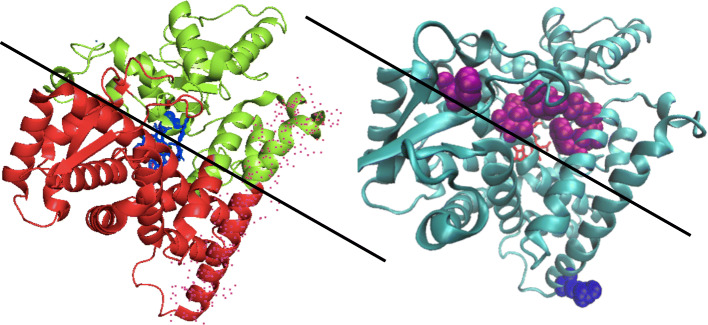
in the left side of the figure the two clusters (red and green) identified on the basis of the contact network analysis applied to the 4kq8 pdb file are reported (the heme group is also shown in blue). The G-helix has been highlighted by a red dot cloud. On the right side, the same structure is reported together with residue R264 (blue, VDW) and those aminoacids (purple, VDW) which form the access channel for the substrate androstenedione (according to Sgrignani and Magistrato, 2012) and the heme group (in red). The black line in both pictures marks the frontier of the two clusters

A topological characterization of the aromatase structure has been attempted using the available crystallographic model of substrate-containing aromatase (PDB code 4kq8), and an in silico version of the ligand-free protein, obtained through a molecular dynamics simulation [25]. A contact network approach consists of a coarse-graining algorithm that reduces the amino acids of a protein into “nodes” of a tridimensional grid, identifying the position of each residue with that of its α-carbon. Each couple of amino acids distance is than evaluated and an (N x N) matrix built, consisting in “1” and “0” elements, corresponding to interacting or non-interacting elements of the network (according to a discriminating distance, fixed a priori). Eventually, “modules” are identified within the protein structure, thanks to a clustering procedure that groups nodes on the basis of the number of their mutual interactions. In the case of aromatase, such analysis allowed the identification of two distinct regions of the protein, as shown in Fig. 7 left panel, where each module of the crystal structure has been highlighted with a different color, red and green, respectively.

**Fig. 8:**
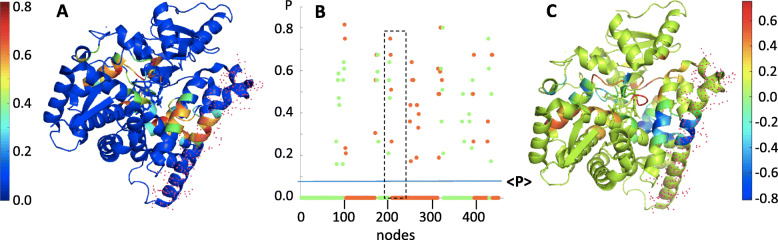
in panel **a** the participation coefficient, P, of each amino acid of wt-Aro is shown, according to the colors code whose numerical value is reported in the left scale. In panel **b** the distribution of the *P* values is presented as a function of the sequence position (X-axis) and of the belonging to one of the two clusters (red or green) identified in Fig. 7. <P > is the protein average participation coefficient. The area of the dashed rectangle corresponds to the G-helix residues. In panel **c** the difference ΔP = P_sb_– P between the P values of the holo and apo forms is reported. The green color has been attributed to those residues whose P value does not change (ΔP = 0) upon ligand binding

The clustering application does not result into an evident domain partition (such as regions with a preponderant kind of secondary structure or areas characterized by specific biological activity), nor the two clusters part in two distinct sections of the sequence. The spatial distribution of each one of the two clusters is uniform and the border may be approximated by a plane perpendicular to the heme group. As shown in Fig. 7 rigth panel, such an abstract plane is placed just below the channel used by the substrate to reach the catalytic site [26] indicating that the boundary between the two clusters is a critical region for the protein biological function. This feature can be more quantitatively described evaluating the so-called “participation coefficient”, P, a parameter that reflects the tendency of each node contained in one cluster to establish connections with nodes belonging to the other group [11]. Such connectivity index ranges from 0 to 1 (Fig. 8a) and the average protein value, <P>, calculated on the whole sequence, results to be <P > ≈ 0.076 (Fig. 8b). Much higher *P* values characterize, instead, the residues lying at the clusters border, including those that form the central section of helix G (dashed rectangle, Fig. 8b). The participation coefficient has been also evaluated in the case of the substrate-bound aromatase model, P_sb_, and the difference obtained between the holo- and apo-form, ΔP = P_sb_– P, is represented in Fig. 8c.

While the majority of the protein residues is not affected by the ligand binding process, the topological analysis suggest that a significant increase in the participation coefficient occurs in the case of those residues that form the binding channel (Fig. 8c). The central section of helix G displays, instead, an opposite effect characterized by the largest decrease in the P value, suggesting that the presence of the ligand reduces the connectivity role that that region has in the open, ligand-free protein form.

## Discussion

Polymorphism of human proteins is a well-known phenomenon [27] that has recently raised a large interest in the scientific community, due to the possibility that cancer predictive studies (based on enzyme variants analyses) have in precision medicine [28]. The systematic study of proteins’ variants databases through new algorithms [29, 30] has considerably improved the accuracy of cancer survival predictions [28, 31–33], giving insights on drugs-induced damages [34] and allowing the identification of new, promising biomarkers [35]. In this general framework, the structural and functional characterization of aromatase polymorphisms is particularly important, as this enzyme is considered as a potential target for breast cancer therapy [36].

The crystallographic structures of placental [37] and recombinant [38] aromatase have demonstrated that the protein has a compact, globular fold and that R264 is localized on the protein surface, far away from the substrate binding site. Despite such a peripherical location, the conversion of ARG to HIS or CYS is known to influence the protein functional properties, reducing its ability to bind the substrate and decreasing its catalytic efficiency [6]. Circular dichroism spectroscopy showed that the two polymorphic variants retain the overall wt secondary structure, but are characterized by a lower thermal stability suggesting that the mutation might change the motility of the helix G to which R264 belongs [6]. Structural studies and molecular dynamics simulations of bacterial P450 have revealed that such alpha helix is involved, through the adjacent helix F, in the opening of the channel which provides the access of substrate to the active site [26, 39, 40].

The contact network analysis reported in the present study has identified two main clusters (Fig. 7), which are “communicating” through residues mainly dislocated along the channel that allows the entrance of the substrate in the active site. Such topological analysis thus suggests that large conformational changes characterize the substrate binding process of aromatase, involving long-range interactions between the two modules. Helices F and G are no exception: their central sections contain a relevant number of crucial nodes connecting the two clusters (Fig. 8a and b) and undergo the largest (negative) change in their participation coefficient, P, as the protein binds the substrate molecule (Fig. 8c). The kinetics of H/D exchange in Aro wt (Fig. 2a and Table 1) indicates that these conformational changes produce a large decrease in the fractionof the more exposed protons in the presence of both androstenedione and anastrozole. Trivial explanations (such as a screening effect exerted by the ligands) seem to be excluded by fluorescence anisotropy measurements, that demonstrate how in the ligand-bound form the protein assumes a more compact tertiary structure through a conformational change that confers the molecule a faster rotational dynamics (Fig. 5). The results obtained for the two polymorphic variants demonstrate that these samples undergo much smaller changes in the H/D kinetics (Table 1) and in the long rotational correlation time (Fig. 5). More importantly, in the presence of both ligands, they retain a higher ANS binding capability, with respect to that of Aro wt (Figure and Table 2), confirming that they lack that compactness which characterize ligand-bound Aro wt. As a consequence, the correct entrance of the substrate (or inhibitor) molecule to the protein active site is compromised, as demonstrated by dynamic fluorescence measurements. Indeed, in a previous study [8], we have demonstrated that the lifetime component at 4.5–5.0 ns can be attributed to W224, since it is not present in mutants such as W224F [8]. Since this tryptophan resides in the proximity of aromatase binding site and it is dramatically quenched by the entrance of androstenedione or anastrozole (Fig. 3a), it can be used to monitor ligand-induced structural changes in the specific region of the active site. The absence of any significant difference of this specific fluorescence lifetime component in the two polymorphic variants (Fig. 3b,c) demonstrates that the local perturbation introduced at the extremity of helix G upon the substitution of R264 impairs the correct placement of the ligand in the active site, thus providing a structural rationale for the decreased activity of the mutants [6].

The normal mode analysis of aromatase [25] has demonstrated that the protein displays a very rigid core (corresponding approximately to the heme group), which is surrounded by concentric shells of progressively more flexible regions. The helices F and G and their inter-connecting loop are one of such external mobile structural areas [38]. Indeed, long-range effects connected to the flexibility of helix G has been already reported in the case of bacterial cytochromes P450 [41]. In particular, it was demonstrated that the breaking of the salt bridge which involves ASP 251 has crucial consequences on ligands recognition and binding.

According to the contact network analysis reported in this study, the protein could be figured as a sort of sandwich, the central slice corresponding to the substrate channel (Figs. 7 and 8). In this picture, the packing of the sandwich would be controlled by the clamping effected exerted by helices F and G, whose hinge mechanism resides in their highly inter-connected central elements (Fig. 8). The substitution of R264 with residues characterized by a different geometry (HIS and CYS), length and charge (CYS) must severely affect the salt bridge interaction with ASP 251, altering the “pivot” mechanism by which the flexible F-G group controls the interconnection between the two clusters.

## Conclusion

In conclusion, the data reported in this study demonstrate by two independent spectroscopic techniques that human aromatase assumes a more compact tridimensional conformation upon ligand binding. On the contrary, mutants R264C and R264H (that impair the correct binding of both substrate and inhibitor molecules) undergo a less efficient packing process, suggesting a minor flexibility of their mobile segments. A rationale to such behavior could be envisaged thanks to a contact network analysis of the protein structure, which has revealed the connecting role of helices F and G between the two clusters that form the substrate channel to the active site. Thus, based on in vitro measurements, such an explanation shed new light on molecular impact that the two polymorphisms have in living beings*.*

## Methods

### Materials

Recombinant human aromatase was expressed in *E. coli* and purified as previously described [7, 8]. Both wild-type and mutants were purified with the same type of buffer, i.e. 100 mM phosphate buffer pH 7.4 containing 20% glycerol, 1 mM β-mercaptoethanol, 0.1% Tween-20.

All chemicals used for protein purification were purchased from Sigma–Aldrich (St. Louis, MO USA) and were analytical grade.

Proteins (wt and mutants) concentration for all fluorescence experiments was about 6.5 μM, while if present, androstenedione and anastrozole concentrations were 20 μM and 10 μM, respectively.

### H/D exchange kinetics experiments by ATR-FTIR

Kinetics of H/D exchange was followed by FTIR as reported by Di Nardo et al. [8]. Experiments were performed at room temperature using an infrared spectrophotometer Bruker Model Tensor 27 (Bruker Instruments, USA) coupled with an attenuated total reflectance (ATR) sampling tool (Harrick Scientific Products, USA). The working parameters were set as follows: scan velocity 10 kHz; resolution 4 cm^− 1^; spectra acquisition frequency limits 4000 and 800 cm^− 1^. Protein sample was analysed by depositing a thin protein film (30 μL, 50 μM) directly on ATR germanium crystal. In particular, Aro was analysed in ligand free form and in ligand bound form, obtained by incubating the enzyme with androstenedione as substrate or anastrozole as inhibitor. The correct binding of both substrate and inhibitor to the active site of Aro was examined by UV/vis spectroscopy by monitoring a shift of the maximum absorbance from 418 nm to 394 nm for Androstenedione and from 418 nm to 422 nm for anastrozole. During acquisition, the spectrophotometer sample chamber was continuously purged with D_2_O enriched nitrogen. Spectra were collected at intervals of 1 min, for the first 10 min, and every 8 min for the following 160 min. For each time point 60 scans were collected and averaged. Spectra were collected at least in quadruplicate and averaged for each time point, corrected by the contribution of control sample, represented by protein storage buffer, and normalized. The H/D exchange kinetics was studied by following the absorbance increase at about 1460 cm^− 1^, corresponding to the shift of the Amide II band upon deuteration. The relative absorbance values were plotted as function of time, fitted to a double exponential function and the deuteration rates were calculated and compared by ANOVA statistical analysis using SigmaPlot software.

### Fluorescence measurements

Fluorescence spectra were collected using a K2-ISS (ISS, Inc.,Champaign, IL, USA) photon-counting fluorimeter thermo-stated at 20 °C using an external bath circulator. ANS binding was studied measuring the fluorescence emission spectra from 450 to 550 nm of the probe using an excitation wavelength of 350 nm. All spectra were corrected by blank subtraction.

8-Anilino-1-naphthalenesulfonic acid (ANS) binding curves were analyzed assuming a simple binding equilibrium, namely, ANS + P ↔ ANS-P, and fitting the total fluorescence, F, at increasing concentration of ANS, [ANS], according to:

$$ \mathrm{F}={\mathrm{F}}_{\infty}\left(\left(\left[\mathrm{ANS}\right]+{\mathrm{P}}_0+{\mathrm{K}}_{\mathrm{d}}\right)-\sqrt{{\left(\left[\mathrm{ANS}\right]+{\mathrm{P}}_0+{\mathrm{K}}_{\mathrm{d}}\right)}^2-4\left[\mathrm{ANS}\right]{\mathrm{P}}_0}\right)/\left(2\ {\mathrm{P}}_0\right) $$

where P_0_ is the total aromatase concentration and K_d_ the dissociation constant of the process.

Lifetimes and dynamic fluorescence anisotropies were performed using the phase shift and demodulation technique on a KOALA-ISS fluorimeter. The excitation source was a 300-nm laser diode, and the emission was collected through a WG320-nm cutoff filter to avoid scattering. Anisotropy decays were collected through Glan-Thompson polarizers taking into account the G-factor correction. All measurements were repeated in triplicate to obtain a good statistic, using a set of at least 30 frequencies. The data have been analyzed with the software provided by ISS.

### Contact network analysis

Protein contact networks (PCNs) rely on a minimalist perspective on protein structures, seen as networks of active contacts between residues (network nodes are the protein residues, and the active contacts between pair of residues are the network links). The definition of active contacts is crucial in defining the properties emerging from the network analysis. In this work, we define “active contact” any contact corresponding to a distance between residues comprised between 4 and 8 Å. This choice includes only significant noncovalent bonds, sensible to the environmental cues. The distance between residues is computed starting from the coordinates of the residues’ *α*-carbons.

The mathematical description of PCNs is provided by the adjacency matrix, defined as:

$$ {A}_{ij}=\Big\{{\displaystyle \begin{array}{cc}1& if4\overset{o}{A}<{d}_{ij}<8\overset{o}{A}\\ {}0& otherwise\end{array}}\operatorname{} $$

Once the network has been built up, it is possible to derive several network descriptor. The most important and simple is the node degree *k*_*i*_, defined as:

$$ {k}_i={\sum}_j{A}_{ij} $$

Based on the node degree, we applied a spectral clustering algorithm to partition PCN into clusters. We applied the method to the network Laplacian, defined as follows:

$$ \boldsymbol{L}=\boldsymbol{D}-\boldsymbol{A} $$

A being the adjacency matrix and D the degree matrix, i.e. a diagonal matrix whose diagonal is the degree vector. The eigenvalue decomposition is applied to the laplacian L: the eigenvector corresponding to the second minor of eigenvalue v2 is of interest for the clustering partition. Considering the partition in two clusters, for instance, nodes are divided into the two clusters according to the sign of the corresponding components of the vector v2.

The partition is binary and hierarchical; the number of cluster (power of 2) is an input value.

In this work, we partitioned the structures into two clusters, so we applied just once the spectral clustering algorithm to the PCNs.

Once the network clustering has been applied, it is possible to derive a clustering descriptor, the participation coefficient Pi, for the i-th residue (node) defined as:

$$ {P}_i=1-{\left(\frac{k_{si}}{k_i}\right)}^2 $$

*k*_*si*_ is the i node degree including only links with nodes belonging to the same cluster s and *k*_*i*_ the node i degree. Thus, P addresses the role of nodes in signal transmission between different clusters (domains in PCNs).

## Data Availability

Not applicable.

## Abbreviations

SNPs: Single nucleotide polymorphisms
Aro: Recombinant human aromatase
FTIR: Fourier transform infrared spectroscopy
ATR: Attenuated total reflectance
ANS: 8-Anilino-1-naphthalenesulfonic acid
PCNs: Protein contact networks
